# Integrative metabolomics as emerging tool to study autophagy regulation

**DOI:** 10.15698/mic2017.08.584

**Published:** 2017-07-13

**Authors:** Sarah Stryeck, Ruth Birner-Gruenberger, Tobias Madl

**Affiliations:** 1 Institute of Molecular Biology and Biochemistry, Center of Molecular Medicine, Medical University of Graz, 8010 Graz, Austria.; 2 Research Unit for Functional Proteomics and Metabolic Pathways, Institute of Pathology, Medical University of Graz, 8010 Graz, Austria.

**Keywords:** metabolomics, autophagy, metabolites, aging, nuclear magnetic resonance, mass spectrometry

## Abstract

Recent technological developments in metabolomics research have enabled in-depth characterization of complex metabolite mixtures in a wide range of biological, biomedical, environmental, agricultural, and nutritional research fields. Nuclear magnetic resonance spectroscopy and mass spectrometry are the two main platforms for performing metabolomics studies. Given their broad applicability and the systemic insight into metabolism that can be obtained it is not surprising that metabolomics becomes increasingly popular in basic biological research. In this review, we provide an overview on key metabolites, recent studies, and future opportunities for metabolomics in studying autophagy regulation. Metabolites play a pivotal role in autophagy regulation and are therefore key targets for autophagy research. Given the recent success of metabolomics, it can be expected that metabolomics approaches will contribute significantly to deciphering the complex regulatory mechanisms involved in autophagy in the near future and promote understanding of autophagy and autophagy-related diseases in living cells and organisms.

## INTRODUCTION

Metabolomics is the key discipline for systemic characterization of the repertoire of small molecules (metabolites) and complements the other ‘omics’ such as genomics, transcriptomics, and proteomics 1,2. The metabolome provides a snapshot of the functional endpoint of complex biological networks and accurately describes the functional and physiological states of an organism 3,4,5,6. Aiming at in-depth characterization of complex metabolite mixtures, the recent technological developments in the field of metabolomics have opened up a wide range of research fields in biological, biomedical, environmental, agricultural, and nutritional research 7,8. In biomedical research, metabolomics has established itself as a key technique for systems biology, disease diagnostics, and biomarker discovery 2,9,10,11,12.

Hallmarks of technological developments that enabled metabolic research and are driving increasingly wider applications of metabolomics were the establishment of powerful analytical instrumentation and, in particular, tools for automated statistical data analysis. Currently, nuclear magnetic resonance spectroscopy (NMR), and mass spectrometry (MS) are the key techniques for the detection and identification of metabolites 2,12,13. Both techniques are complementary: on the one hand NMR provides access to unique structural information, is quantitative and highly reproducible, providing that guidelines for sample preparation and experimental setup are followed 14,15,16,17,18, but less sensitive 19,20,21. On the other hand, MS is more sensitive than NMR, but suffers from the ambiguity of spectral signatures. The complementary nature of NMR spectroscopy and MS for metabolomic analysis has been impressively demonstrated in several studies 15,22,23, suggesting that the combination of both techniques is beneficial for a more comprehensive metabolite identification than applying each platform alone.

This review focuses on recent developments in the field of metabolomics with a particular emphasis on the integration of NMR spectroscopy, MS, and data analysis methods for revealing the complex regulatory mechanisms involved in autophagy. This is, to our knowledge, the first review with a particular focus on integrating MS- and NMR-based metabolomics research for autophagy-related studies. By extending the currently available toolbox in autophagy research with recently developed and powerful metabolomics and data analysis approaches, we anticipate that new mechanistic insights into the regulation of metabolism in autophagy can be obtained. The main aim of this review is to introduce MS-, and NMR-based metabolomics to an audience of scientists with a biological focus on autophagy. With this foundation, recent static and dynamic studies of metabolite networks involved in autophagy will be discussed. By establishing metabolomics as a general approach in autophagy studies, unprecedented opportunities will be opened up for scientists with a biological focus on autophagy in terms of exploration of the metabolome for markers of disease states, and in understanding the diversity of metabolic pathways of autophagy in a variety of organisms. The knowledge gained from this approach provides a ready link to genomic, transcriptomic, and proteomic information to achieve systems biochemical understanding of autophagy in living cells and organisms.

## AUTOPHAGY AND METABOLISM

Autophagy is a self-degradative process balancing synthesis and degradation. It is a process disassembling unnecessary or dysfunctional cellular components. First studies revealing intracellular protein degradation and lysosomes provided important fundament for discovery of autophagy 24 which has initially been described in eukaryotes 25,26. However, similar processes are observable in all microbes, including bacteria 27, archaea 28 and most protozoa 29. These processes include bacterial cannibalism, autolysis, programmed cell death and other self-destructing patterns 25,30. This balance is mediated via degradation of cytosolic proteins and organelles in order to maintain cellular function 31. In case of lack of resources of vital importance, cells boot up their adaptive response to the environment, namely autophagy, to ensure proper supply of molecular building blocks in order to synthesize limiting essential components. Three distinct pathways of autophagy are described in the literature comprising the main pathways: macroautophagy 32, microautophagy 33, and chaperone-mediated autophagy 34. All of these autophagic pathways pursue the same goal: providing essential compounds to ensure proper cellular function. However, the underlying regulatory mechanisms are different.

Microautophagy degrades cytoplasmic components via lysosomal shuttling 33. Chaperone mediated autophagy leads to regulated transport of cytoplasmic proteins into the lysosome and their subsequent lysosomal degradation. This type of autophagy is depending on molecular chaperones 34. A detailed discussion of micro-, and chaperone-mediated autophagy can be found elsewhere 34,35. In this review we focus on the metabolic processes involved in macroautophagy. Macroautophagy, hereafter referred to as autophagy, is an inevitable physiological process, ensuring quality control of proteins and organelles in order to maintain cellular homeostasis. It acts, on the one hand, as a cellular housekeeper under normal physiological conditions and, on the other hand, as an inspector participating in the clearance of protein aggregates and improperly functioning organelles, which is a hallmark of aging 36,37,38. Besides of these essential control functions, autophagy is indispensable for developmental processes. In line with this, autophagy-deficient mutants lack the ability to modify intracellular architecture and rapid response to external cues and show developmental impairment 39.

Autophagy is mediated by formation of transient double-membrane structures, the so-called phagophore. The phagophore becomes an autophagosome after expansion and closure, fuses with lysosomes, and degrades targeted organelles via acidic hydrolases 40. Since autophagy is a complex process, a plethora of proteins is involved in the regulation of the autophagic processes. The main key players are the so-called autophagy related (Atg) proteins 41. The core machinery of Atg proteins comprises around 30 members, which mediate processes from early autophagosome formation, including unc-51-like kinase/autophagy-related 1 (ULK/Atg1) and phosphoinositide 3-kinase (PI3K) complex formation to subsequent stages of vesicle elongation and completion 35,42,43.

Autophagy is a protective response; however, it is closely linked to the cell death program. If autophagy, as a primary response to cellular damage, fails, it gets blocked and apoptosis is induced. Inhibition of autophagy may be a consequence of caspase-mediated cleavage of Atg proteins and binding of the pro-apoptotic molecule Bim to Beclin 1, a member of Atg proteins 44. Autophagy and cell death can also coexist. For instance, in ferroptosis, an iron-dependent form of regulated necrosis, autophagic degradation of cellular iron storage proteins plays a crucial role 44,45.

Given the involvement of autophagy in various physiological aspects, it is not surprising that autophagic processes have to be tightly regulated as an adaptive response to unfavorable conditions. Autophagy-inducing cellular stress conditions include starvation and mechanical stress 46, but also endoplasmic reticulum stress, growth factor deprivation and pathogen infection 47. Levels of glucose, amino acids, and lipids reflect nutrient supply and cellular energy status and constitute the pivotal metabolic regulatory factors.

This review focuses on metabolites involved in autophagy regulation, their investigation using metabolomics methods, applications and future opportunities of metabolomics in revealing key regulatory mechanisms in autophagy.

Due to the general importance of metabolites, including amino acids, carbohydrates, and lipids, in autophagy, metabolomics is well suited to study autophagic processes. Up- or downregulation of specific compounds reveal regulatory processes and, combined with other ‘omics’ data, give a versatile molecular insight into autophagy. Regulation of autophagy can be studied by modern metabolomics techniques in a variety of different matrices, including cell lysates, tissue lysates and all types of biofluids. By studying different model systems, including genetic variants of proteins, knockdowns of proteins or application of pharmacological inhibitors, effects of autophagy can be studied and evaluated 48,49. Through this, metabolomic studies provide insight into key regulatory mechanisms of autophagy and autophagy-related processes.

## SETUP OF METABOLOMICS STUDIES

**Figure 1:**
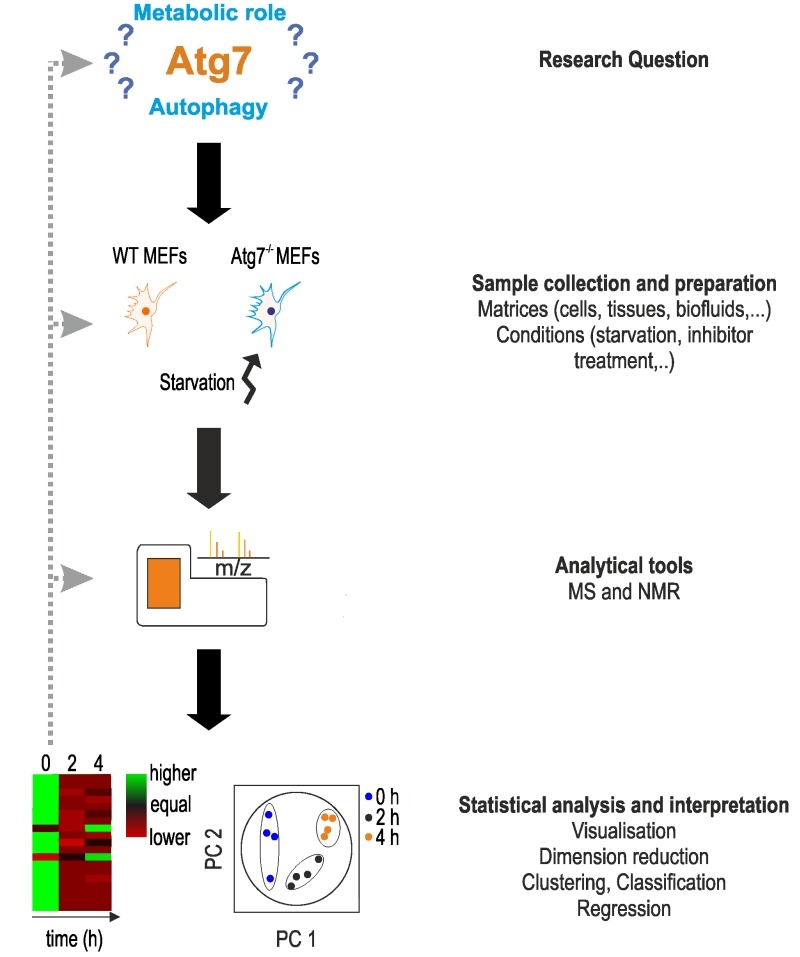
FIGURE 1: Characteristic setup of a metabolomics study. The setup of a metabolomics study is shown using the work of Shen *et al*. as an example 52. Main steps are shown on the right. The main aim of the authors was to determine the function of Atg7, a protein essential in the formation of autophagosomes. In absence of Atg7, cells indicate a reduction or lack of autophagy, however, the metabolic responses under acute starvation remained elusive. The main aim of the study was to determine the metabolic phenotype of Atg7-dependent autophagy under starvation. Based on this research question, the authors used wild-type and Atg7^-/-^ mouse embryonic fibroblasts (MEFs) as a model system and starvation as experimental conditions. Examples for other material and conditions can be found on the right. After treatment, cells were lysed for metabolomics analysis on an UPLC-MS system. Apart from MS analysis, NMR spectroscopy can be applied. Metabolomics data were analyzed statistically using principle component analysis (PCA), and heat maps were used to visualize changes in metabolite concentrations. For more details, see text.

Metabolomic studies, including autophagy-related metabolomics, follow a certain general scheme 15,50,51 (Fig. 1).

The first step is the verbalization of a specific biological research question. This question provides fundamental information needed for the setup of the metabolomics approach, and includes decisions on whether the study is targeted or untargeted, sampling of material, sample preparation, the analytical technique, and data analysis. In an untargeted approach, a general profile of metabolites present in the biological sample is obtained. This is mainly used to detect global differences in the metabolic fingerprint 11,53. For targeted analysis, the experimental setup is optimized for the detection of distinct metabolites 54. Sampling of appropriate biological material is critical to answer the research question. A broad range of biological material, ranging from cell-based material, *in vivo* model systems, and human samples, can be used for metabolomics studies. Examples include, but are not limited to, cells, multi-cellular model systems (e.g. organoids 55), cultivation media, tissues, and biofluids (blood, cerebrospinal fluid, urine, feces), respectively. These studies can be carried out in combination with any kind of modulations, such as pharmacological compounds or genetic modifications (knock-ins/outs, CRISPR/Cas9 technology) 49,54,56. Care has to be taken that proper controls are selected, that samples are taken using standardized operating procedures (SOPs), and that samples are stored under suited conditions. Inappropriate sample storage or preparation builds the main source for errors in metabolomics studies. For instance, a different storage of samples may change the entire metabolic profile due to aberrant enzymatic quenching at different temperatures or different stabilities of metabolites 57,58. Furthermore, an appropriate sample size needs to be selected to ensure statistical significance of the results. In clinical studies in particular, the biological variation in patient samples can be high due to environmental factors, but also nutrition and genetic loading 59,60. Particularly in untargeted approaches neither the number of analytes nor the effect size are known a priori, which makes an estimation of a required sample size difficult. However, it can be estimated, which ranges can be covered and which information can be obtained with a larger sample size 61.

Sample preparation is often necessary for metabolomics studies, and typically includes lysis and/or extraction steps. The aim of these procedures is to release metabolites, to remove interfering substances (e.g. insoluble components, proteins, lipids), and to optimize sample stability, such as by quenching enzymatic turnover of metabolites after lysis. Lysis and extraction protocols are tailored to the metabolite of interest, the matrix and the analytical technique used for metabolic profiling 40. Most commonly used protocols to break cells and tissue employ lysis by sonication or use of a bead homogenizer 32,49, combined with an extraction procedure with a solvent mixture (e.g. H_2_O/MeOH or H_2_O/MeOH/CHCl_3_) 52. For certain tissues it might be necessary to grind them frozen with liquid nitrogen. Protein removal might be carried out alternatively using ultrafiltration 16. Internal standards can be included in the extraction. However, it must be ensured that these standards remain in the solvent, i.e. do not interact with sample components, which are removed during the extraction (e.g. protein precipitate). Examples where lysis or extraction steps are omitted are solid state NMR spectroscopic studies of intact tissue and solution NMR spectroscopic studies of urine or cerebrospinal fluid 7,54. In case of MS, samples are measured either directly from the matrix or using extraction protocols to enrich the metabolites of interest 15. Furthermore, derivatization techniques are used to improve analytical behavior of metabolites. Examples are the increase of stability or volatility of non-volatile or instable compounds in gas chromatography 62, or removal of metabolites due to interaction with cationic or anionic silica nanoparticles 63, and introduction of ^15^N using a cholamine tag in NMR spectroscopy. This latter tag binds to the carboxyl group of metabolites and introduces on the one hand ^15^N as a second stable isotope, which is NMR active, and on the other hand a permanent charge, which is detected by MS 64. Difficult-to-be-ionized metabolites are not easily measured by MS, but ionization can be improved by chemical derivatization such as isotope coded derivatization (ICD) 65. For normalization of metabolite concentrations in cell lysates and tissues, protein concentrations and tissue (dry) weight are most commonly used. Other approaches include DNA concentration in adherent cell lines 66, or total ion current in MS, or total integrated proton signal in NMR 67. In certain matrices metabolites can be normalized to an endogenous substance, such as creatinine in urine. However, care has to be taken that concentrations of these metabolites are not affected by diseases, such as renal injury in case of creatinine in urine.

After preparation, samples are investigated on a dedicated analytical platform. In NMR spectroscopy-based metabolomics, the platform is a NMR spectrometer, whereas in MS-based metabolomics, the analytical platform can comprise, besides the mass spectrometer, an additional chromatographic separation step. Depending on the research question, the profiling approach, and the type of metabolites of interest, liquid or gas-chromatography (LC or GC) respectively is used. LC is applied to the majority of chemical species, typically without any chemical modification of metabolites, whereas GC requires derivatization to improve volatility and thermal stability of polar compounds (such as amino acids, organic acids, sugars, amines, alcohols and amides). However, GC achieves a better metabolite separation due to its higher resolution and generally reduces matrix effects and ion suppression 22,68,69. On the other hand, HPLC, and the higher efficiency variants, such as U(H)PLC, offer the most versatile tools for the analysis of a multitude of molecules which belong to different groups, have different molecular properties and coexist in the same sample in varying concentrations. Typically U(H)PLC is used, but nano LC and "chip"-based LC may also make a contribution in this area 70. Depending on the metabolite of interest, LC-MS typically uses reverse-phase chromatography for apolar compounds, whereas hydrophilic interaction liquid chromatography (HILIC) is chosen for polar metabolites 71,72. For polar and charged metabolites, ion chromatography 73 and capillary electrophoresis 74 have also been used. Supercritical fluid chromatography (SFC), using CO_2_-based mobile phases, is an alternative separation tool and may have great utility for certain applications such as lipidomics 75. In principle, NMR spectroscopy can be coupled to liquid chromatography, but this approach is not applied routinely 23.

For targeted MS analysis, internal standards are often spiked into the sample, enabling an absolute quantification of metabolites of interest 68,76. In NMR based metabolomics, targeted or untargeted approaches use similar experimental setups, and spike-in of reference material is typically used to verify certain metabolites at the late stage of analysis.

From a physical point of view, NMR spectroscopy and MS provide fundamentally different experimental and therefore complementary metabolite data. NMR spectroscopy determines resonance frequencies of NMR active nuclei (chemical shift), most commonly ^1^H, ^13^C, ^15^N, and ^31^P, with the intensity of each signal being determined by the concentration of the corresponding metabolite. MS provides mass-over-charge ratios of each metabolite and its adducts as well as its fragments in the case of tandem mass spectrometry, with the signal intensity being determined by the concentration and ionization property of the corresponding metabolite (and for the fragments additionally their fragmentation efficiency) 51.

MS further benefits from using the aforementioned chromatographic techniques as frontends. Coupling the MS analyzer to chromatography comes with several advantages: on the one hand, matrix effects and ionization suppression are reduced and isomers and isobars are separated, which reduces spectral complexity, improving metabolite identification and quantification. On the other hand, more information is obtained due to orthogonal data obtained in chromatography (i.e. retention times), also improving identification. However, these approaches are more time-consuming than direct infusion MS and might complicate trouble-shooting, which may build an obstacle in high-throughput analysis 77. Utilization of MS in the direct infusion mode has been very helpful in high-throughput quantification of metabolites in complex mixtures, in which case very high mass accuracy is a prerequisite. However, if a chromatographic separation is not applied, an unknown number of molecules of unknown properties and concentrations are subjected simultaneously to the ionization process and thus poor ionization efficiency will be observed for numerous analytes. MS can also be coupled to ion mobility spectrometry, which provides information about drift times of ionic molecules in the gas phase dependent on their shape, supporting metabolite identification and improving peak capacity for MS-based quantitation by adding another dimension 78.

Electrospray ionization (ESI) is the preferred ionization mode because it is easily coupled with LC and typically profiles are obtained in both positive and negative ion mode. So far, the majority of MS-based global metabolite profiling studies have been realized using a combination of U(H)PLC with time-of-flight mass spectrometry (TOF-MS) 70. Such systems combine the highest chromatographic resolution with excellent sensitivity, fast data acquisition and high mass accuracy. Higher resolution MS machines (e.g. FT-ICR) typically require longer times to achieve higher resolution, hence, such instruments do not fully exploit the potential of fast UHPLC. The Orbitrap-MS is also widely used in metabolomics research, either as standalone MS e.g. for direct infusion, or with matrix assisted laser desorption ionization (MALDI) for MS-imaging, or combined with various modes of LC. Orbitraps offer very high resolution and mass accuracy and MSn capabilities. High mass accuracy is very useful for the identification of metabolites: more precise atomic composition data is attained, thereby reducing the number of candidate identities 79. The combination of high mass accuracy MS and MS/MS data with library searching, the use of authentic standards and information from other experiments (e.g. NMR) gives a much higher level of structure identification/confirmation ability.

NMR spectroscopy-based metabolomics is a non-destructive method, which enables performing complementary NMR experiments on the same sample. Hereby, different NMR pulse sequences can be used to optimize the information content of the NMR data. The obtainable information ranges from chemical shifts, scalar couplings, connectivity, spatial proximity, diffusion properties, to the isotope content. These experiments are either recorded as one-dimensional experiments or higher dimensional experiments where several information contents are combined, such as ^1^H chemical shifts and connectivity in 2D ^1^H-^1^H Total Correlation Spectroscopy (TOCSY), or ^1^H and ^13^C chemical shifts and connectivity in 2D ^1^H,^13^C Heteronuclear Single Quantum Correlation (HSQC) type NMR experiments. The purpose of higher dimensional experiments is to reduce signal overlap, to provide complementary information for identification of (yet unknown) metabolites, and to enable analysis of isotope incorporation in metabolic flux analyses 2,15,80.

NMR spectroscopy and MS are two complementary techniques, which, in combination, provide the outmost information content for metabolic studies 13. NMR-based metabolomics is typically used as an untargeted approach, providing a plethora of metabolite information. The main limitations in NMR-based metabolomics encompass the lower information content for apolar samples, as well as the lower sensitivity, which requires sample concentrations in the low micromolar range. MS provides complementary information to NMR spectroscopy by application to lipidomics, and is the method of choice for low concentrated compounds due to its high sensitivity (picomolar to femtomolar). MS is a well suited technique for targeted metabolomics, however, it is not straightforward for application to all matrices 13,14,15,21,76,77,80,81. Thus, by combining the two techniques high quality metabolomics data can be obtained in a robust and reproducible way 82.

For targeted metabolomics, metabolites are identified and quantified based on their characteristic chemical shifts and splitting patterns in the case of NMR spectroscopy, and based on their characteristic mass-over-charge ratios, isotopic distribution and retention times in case of LC/GC-MS, and characteristic fragments if tandem MS is employed. The use of multiple reaction monitoring (MRM) mode, available in triple quadrupole MS instruments allows monitoring of selected precursor ion masses, which can be further fragmented into several new fragments, and one or a few of these fragments. The chances for an interfering compound having the same retention time in the column, and having exactly the same m/z, and producing the same fragment are low, which reduces the background noise to close to zero, making MRM mode very specific and sensitive 83. High resolution mass spectrometers (TOF, FT-ICR, Orbitrap) allow pseudo MRM acquisition by monitoring selected precursor ions and obtaining MS/MS spectra of their fragments which can then be used for quantitation (MS2-based quantitation) 84. Data-dependent acquisition (DDA) is widely used for untargeted metabolomics studies employing tandem mass spectrometry (MS/MS). DDA selects precursors for fragmentation by their intensity. Thus, only the most intense ions are fragmented. This affects reproducibility, accuracy and sensitivity of detection and quantification of the analyzed target metabolome across multiple samples. Data-independent MS/MS acquisition methods 85, in contrast, can theoretically obtain all fragment ions for all precursors simultaneously, thereby increasing the coverage of observable molecules and improving analytical reproducibility and quantitative performance. However, the main problem is the complexity of MS/MS spectra because of the wide isolation window (20-50 m/z or more) for precursor ion selection, requiring a high performance MS platform, proper precursor isolation scheme settings, and reliable post-acquisition data-processing 86. The MSE workflow even isolates all precursors in a whole MS/MS scan instead of employing consecutive isolation windows, which can also be called all-ion fragmentation (AIF) strategy 87. For DIA data processing, the acquired original MS/MS spectra consist of fragments of several precursors, and thus need to be deconvoluted to reveal the MS/MS spectra for each precursor 85.

Untargeted analyses can detect hundreds for GC to thousands of molecules for LC-MS-based methods 88. The main limitation of current untargeted LC-MS platforms is the unambiguous identification of the molecules. Generally, identification requires the use of the accurate mass measurement to limit the possible molecular formulas of candidate molecules, matching for retention time and m/z ratio and/or a specific compound fragmentation with a standard compound. Even though the number of metabolites in databases is growing and automated softwares for identification are getting better, there are still many molecules which cannot be identified by database searches. Tandem mass spectral search (MS/MS) from large well established libraries, such as the National Institute of Standards and Technology database 89, the Human Metabolome Database 90 and METLIN 91, is the fastest way to correctly annotate MS/MS spectra from screening small molecules 92. The confidence in MS/MS-based annotation of chemical structures is impacted by instrumental settings and requirements, data acquisition modes, library scoring algorithms, as well as post-curation steps. Methods combining both targeted and untargeted approaches in the same run have also been developed 93,94 to get the best of both approaches.

Metabolite concentrations can be determined simply by integration of the corresponding NMR signal(s), and using an internal or external reference with known concentrations. Most state-of-the-art metabolomics NMR experiments, such as the ^1^H 1D one-pulse sequence, nuclear Overhauser effect spectroscopy (NOESY) and CPMG (Carr-Purcell-Meiboom-Gill) pulse sequences with water suppression using presaturation are quantitative and enable a direct determination of metabolite concentrations in the samples 14,15,18,19,20,21. For absolute quantification in MS, stable-isotope-labeled standards are typically spiked into the sample, to overcome potential bias due to metabolite specific ionization properties 13,21,42,54,56,95. Receiver Operator Characteristic (ROC) curves are often used to further evaluate metabolite concentrations in order to provide information about the discriminatory power of metabolites and metabolite patterns 96.

In untargeted metabolomics, the vast amount of generated data is analyzed using cheminformatics tools. The aim of these tools is to retrieve characteristic patterns in metabolite profiles and a statistical evaluation, thereof, in order to identify for example differences in metabolite profiles, reflecting physiological or pathophysiological metabolic fingerprints 97,98. Since data sets obtained by MS and NMR spectroscopy are complex and involve a high number of data points, it is beneficial to use multivariate statistical approaches in order to avoid loss of critical information 99,100.

A variety of statistical approaches have been reported to be applicable to complex data sets, including unsupervised approaches (i.e. PCA, k-means clustering, hierarchical clustering, Hidden Markov models) to draw interferences from datasets without information of group affiliation and supervised approaches (i.e. PLS, PLS-DA, OPLS-DA), providing information about group affiliation. Two of the most popular approaches for metabolomics studies are PCA and OPLS-DA (orthogonal partial least square- discriminant analysis). PCA is an unsupervised approach, i.e. that it can identify patterns and regularities without direct supervision of a human. The algorithm identifies data points (i.e. chemical shifts, mass-over-charge values, retention times) which show the largest differences between samples, or in other words the metabolites which are varying most between samples. Those are combined in so-called principle components which are projections of the multidimensional variable space and reveal similarities between samples. For evaluation of sample sets and to detect outliers, Hotelling’s T^2^ ellipse provides a measure of the variation in each sample within the model after projection trough the model and indicates how far each sample is from the center of the model. In order to account for most of the data variability, cross-validation can help to find the appropriate number of principal components 101,102. Summarizing, PCA reveals differences and similarities of metabolic signatures between samples and the metabolic signatures causing these differences 52. This allows identification of outliers and clusters of samples with similar metabolite profiles.

In the supervised Partial Least Square (PLS)-based approaches regression models are used for multivariate data analysis to answer what metabolite signature discriminate two groups of samples. To this end, the information of two blocks of variables is compared to each other and the fundamental relations are identified. To further improve the model, orthogonal components, corresponding to variables varying within groups, can be included in the building process of the model (OPLS-DA) 103. To optimize information of this multivariate statistics approaches and to assess the quality of the model, cross validation is used. Cross validation can help to avoid overfitting by partitioning data into subsets and validating their match to the sample set 8,104.

If both, NMR and MS data sets are available, these data can be integrated in the multivariate data analysis using O2PLS. Therein, the principle of OPLS-DA is extended and enables the identification of patterns provided by both data blocks (i.e. NMR and MS) and patterns unique to one block, thus providing an improved interpretation of complex data sets 105. Finally, the integration of data obtained by other complementary techniques, such as proteomics, transcriptomics or genomics in OnPLS statistical analysis, can provide the outmost information content for biological systems 9.

These statistical approaches include complex calculations which implies that automation of data analysis is inevitable. Pipelines for metabolomics data processing and statistics have been setup in several free and commercial software packages and web servers (for recent reviews see 10,31,106). Due to the increasing amount of generated data, other computation approaches such as machine learning and artificial intelligence gain in importance. Machine learning is a powerful tool making predictions based on huge amounts of data using complex algorithms 107. First metabolomics studies using artificial intelligence were published recently. Yuan-yuan Xie *et al*., for example, used artificial neural networks and neurofuzzy logic to predict potential biomarkers in a stress-induced rat model to characterize the therapeutic effects of a Traditional Chinese Medicine using UPLC-QTOF/MS 108. Brougham *et al*. successfully used artificial neural networks in order to classify drug resistance patterns in lung carcinoma cell lines using NMR spectroscopy data 109.

Besides these approaches, which provide a static snapshot of the metabolism at a certain time point, metabolic flux analysis provides detailed dynamic information on metabolic processes. Assume, for example, a case where decreased levels of metabolites are observed. Two alternative mechanisms might lead to this observation which cannot be discriminated: either increased metabolite consumption or decreased production. Moreover, metabolism provides alternative pathways, which may converge on the same metabolite. By administering isotope labeled compounds, for example ^13^C-labeled glucose or glutamine, to the model system of interest, it is possible to track the way of metabolites through metabolic pathways and obtain information through which pathway the metabolite of interest has been generated. By analyzing different time points, the decrease of labeled compound, as well as the increased incorporation of isotopes in other metabolites, can be followed by NMR spectroscopy or MS 110. Thus, metabolic flux analysis is a versatile tool to study metabolic regulation, for example in autophagic processes 48,111,112,113. Application of NMR spectroscopy and MS in metabolomics studies thus provides a plethora of information which enables understanding of physiological and pathophysiological processes. In autophagy research, metabolomics is a relatively young technique and has an enormous potential for applications. In general, metabolomics concerns the sum of all metabolites present in a biological system. In the following paragraphs, we will highlight how NMR and MS have already been employed for metabolomics studies of autophagy and conclude with a future outlook.

## METABOLOMICS STUDIES OF AUTOPHAGY REGULATION

Autophagy helps cells to cope with several stresses. Due to the variety of different factors leading to stress situations, it is not surprising that regulation of autophagy is mediated by different pathways and a variety of different proteins and metabolites 34,35,43,47,114,115. To highlight the general applicability of metabolomics to study autophagy, we provide an overview on recent key metabolites and metabolomics studies of autophagy regulation. In addition to physiological regulation of autophagy, metabolomics can provide insight into autophagy-related diseases in cell-based assays, animal models, or humans.

**Figure 2:**
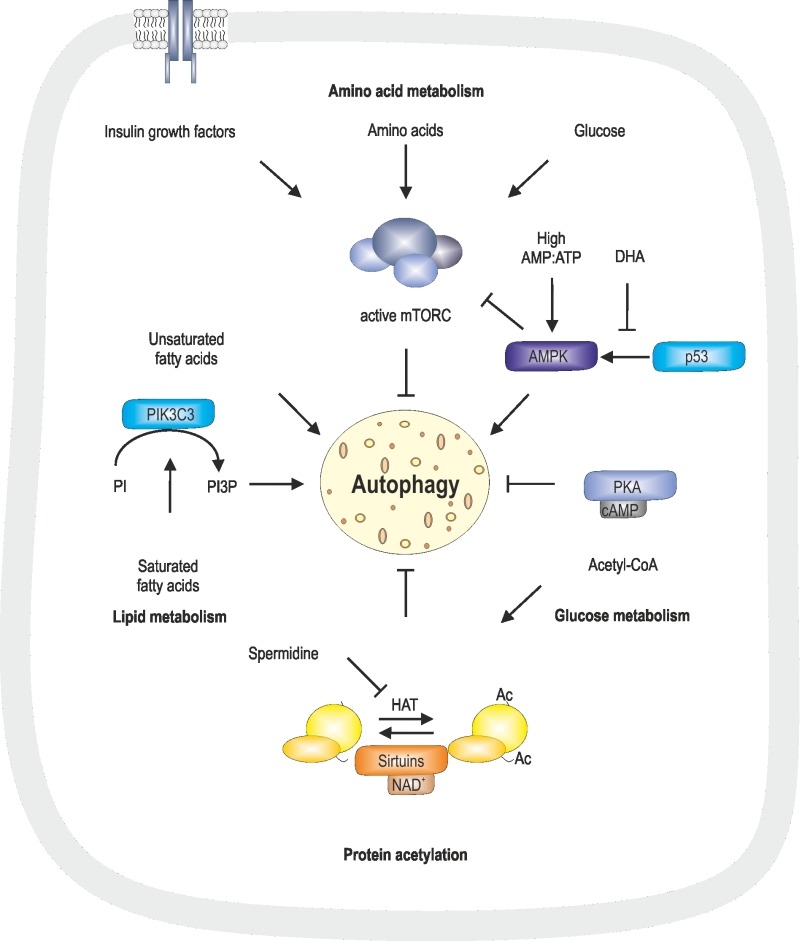
FIGURE 2: Regulatory pathways involved in autophagy. Regulation of autophagy is mediated via two key conditions: starvation, determined by amino acid, glucose and lipid levels, as well as protein acetylation status. Protein acetylation is mediated via HATs, which can be inhibited by spermidine, and deacetylation is mediated via NAD^+^-dependent sirtuins. Acetyl-CoA, a product of glycolysis, is a substrate for protein acetylation. DHA is associated with low p53 levels, which translates into inactive AMPK. In absence of glucose, high AMP:ATP ratios activate AMPK, which, in further consequence, inhibits mTORC and therefore induces autophagy. High glucose levels result in high cAMP levels and therefore active PKA, which inhibits autophagy. mTORC is activated by insulin growth factors, amino acids and glucose and, in its active state, inhibits autophagy. Unsaturated fatty acids are associated with non-canonical autophagy, whereas saturated fatty acids activate PIK3C3, that converts PI to PI3P, which is associated with autophagy. These metabolites involved in regulation of autophagy are discussed in detail in the text.

Induction of autophagy can be mediated by starvation conditions, such as low levels of essential metabolites including glucose and amino acids. Low glucose levels reflect in low cAMP levels and decreased AMP:ATP ratio 115. In addition, insulin-like growth factors, recognized by insulin growth factor receptor, glucose, amino acids and fatty acids lead to an active state of the mammalian target of rapamycin complex (mTORC) and therefore inhibition of autophagy 116. Glucose can further be metabolized to acetyl-CoA, serving as a substrate for protein/histone acetylation, which, at a high level, inhibits autophagy 46,117,118. This modification is inhibited or reduced by metabolites such as the polyamine spermidine and NAD^+^ via NAD^+^-dependent sirtuins 46,118 (Fig. 2).

Since most state-of-the-art metabolomics approaches have only recently been developed and are still developing, the number of studies focusing on autophagy and metabolomics is limited. To avoid any further restriction of the application spectrum of metabolomics to studies in microbes, we present a broad overview of key studies focusing on the use of metabolomics approaches in autophagy research. We apologize for any study that has not been included or which was not discussed in detail due to space limitations.

### Glucose, cAMP, AMP:ATP ratio

Glucose is a key metabolite in autophagy regulation. It reflects the energy state of the cell; if glucose levels are high, cells are usually not in starvation conditions and, under physiological conditions, exhibit reduced autophagy. Carbohydrates, especially glucose, are well-detectable available in metabolomics studies 119,120.

In presence of high glucose levels, ATP is converted to cAMP, which is a cellular measure for nutrient availability, indicating high nutrient levels. Under these conditions, autophagy is inhibited due to elevated levels of cAMP through activation of protein kinase A (PKA), which in turn phosphorylates autophagy-related proteins (i.e. Atg1, Atg13) and mTORC. This leads to the inhibition of the pre-autophagosomal structure (PAS) and reduced autophagy. Low glucose levels result in loss of this inhibitory phosphorylation and activation of autophagy. Taken together, detection of high cellular cAMP levels indicate reduced autophagic flux 115. High glucose levels result in a high energy level which is reflected by an increased ATP:AMP ratio. Absence of glucose translates to low cellular energy levels, namely a low ATP:AMP ratio, which is sensed by AMP kinase, and results in inhibition of the mTORC1 complex, either directly by phosphorylation or indirectly by phosphorylation and activation of tuberous sclerosis protein 1/2 (TSC1/2), which is an inhibitor of mTORC1 116. Furthermore, AMP kinase phosphorylates the protein unc-51-like kinase 1 (ULK1) 50. Thus, glucose, ATP, AMP, and cAMP levels are well-suited readouts for cellular autophagic capacity, with high levels of these metabolites reflecting reduced autophagic flux. Glucose, and nucleotides, if present in high concentrations, are detectable using NMR spectroscopy and MS. cAMP is a signaling metabolite, which may be present in very low concentrations, therefore, highly sensitive MS approaches are well applicable 121,122,123.

Autophagy is a tumor suppressive process, but in case of tumors harboring mutations in Ras, they highly depend on autophagy. In addition, cancer typically depends on high glucose levels 115]. Lashinger *et al*. investigated a mouse model system with respect to the effects of caloric restriction and autophagy on Ras-driven tumors. Given the autophagy-dependency of Ras-driven tumors, the main research question in this study was if, by combining caloric restriction (CR) and autophagy inhibition, the tumor growth might be inhibited more efficiently than using either treatment alone. To investigate this research question, model systems were generated using mice transplanted with Atg5^+/+^ (control) and Atg5^-/-^ (autophagy inhibited) tumor cells. Both, CR and autophagy deficiency, were sufficient to reduce proliferative cells within the tumor; combined CR and autophagy inhibition reduced the tumor volume the strongest. In order to understand the metabolic effects of caloric restriction on tumors four weeks after transplantation, global metabolic profiles of mouse serum were compared by NMR metabolomics. For this purpose, protein was removed by ultrafiltration, protein-free filtrate was mixed with NMR buffer and measured directly. Using PCA as statistical analysis approach and quantification of metabolites of interest, a switch away from glucose metabolism upon CR was observed, indicated by an upregulation of ketone bodies and a downregulation of glucose, amino acids and tricarboxylic acid cycle (TCA cycle) intermediates. Quantification and statistical analysis was performed using commercial cheminformatics software packages 49.

Lock *et al*. performed a complementary study to investigate the relationship between autophagy and metabolism in a murine cell model system 48. Their aim was to delineate the biological contributions of autophagy to Ras-mediated adhesion-independent transformation. In order to answer this research question, they used mouse embryonic fibroblast (MEF) cells as a model system to study autophagy in Ras-mediated transformation. Given the glucose dependency of Ras-tumors, Atg5^-/-^ or Atg5^+/+^ MEF cells were chosen to monitor glucose metabolism by NMR spectroscopy, using [1-^13^C] labeled glucose as nutrient supplement. Metabolites were extracted with methanol/chloroform. The aqueous phase was lyophilized and re-dissolved in D_2_O for NMR measurements. Concentrations were determined using an external reference, and samples were normalized to total cellular protein. To perform a metabolic flux analysis, they followed the metabolic fate of ^13^C-labeled glucose by observing the incorporation of stable ^13^C isotopes from glucose into downstream metabolites. Statistical significance was calculated using analysis of variance (ANOVA). Upon activation of expression of human oncogenic Ras (H-RasV12), decreased levels of [3-^13^C] alanine, which is the product of transamination of the glycolytic end product pyruvate, in MEFs lacking Atg5, were observed. These data suggest a higher glycolytic activity and a higher sensitivity to glucose deprivation in autophagy-competent cells. This study points out the power of metabolic flux analysis for monitoring incorporation of stable isotopes into metabolites and dissection of metabolic pathways.

Redmann *et al*. conducted a study in a murine cell model system to investigate the molecular action of pharmacological inhibitors of autophagic processes, focusing on substances that target lysosomes, but for which the underlying mechanisms are different. Based on the current knowledge about dependency of mitochondrial quality control on autophagy, their main aim was to characterize the influence of the pharmacological inhibitors bafilomycin A1 or chloroquine, on cellular bioenergetics of primary cortical rat neuron cells as model system. To this end, they performed targeted analysis of metabolites of the TCA cycle by HPLC-MS. Samples were prepared by scraping cells in ice-cold methanol to quench enzymatic activity and, after centrifugation, dried supernatant was dissolved in HPLC mobile phase and measured. Due to characteristic mass-over-charge ratios and retention times, various metabolites, including citrate, succinate, fumarate, glutamate, and aspartate, were detected, quantified and subjected to ANOVA. As a result, metabolites of the TCA cycle, particularly those downstream of citrate synthase and those linked to glutaminolysis, were decreased in the autophagy inhibitor treated cells. These results implicate that inhibitors of autophagy impact on cellular bioenergetics and metabolism probably due to decreased mitochondrial quality control. The study exemplifies that metabolomics is a powerful tool for investigation of effects of pharmacological treatment to increase our understanding of regulatory mechanisms of autophagy 54.

### Amino acids

Free amino acids are building blocks of proteins and therefore, are highly concentrated in cells after proteolysis during autophagy. Amino acids can be measured and detected by NMR- and MS-based metabolomics and are therefore good markers for (in)activation of autophagy, depending on the model and experimental conditions used 124,125. As already discussed, glucose, and insulin growth factor receptor are important regulators of mTORC 116. However, glucose alone is insufficient for activation; free amino acids are also required in this process 126,127. In the presence of high amounts of amino acids, mTORC1 is activated and inhibits induction of autophagy by phosphorylating Atg proteins. The mechanism involves H^+^-translocating ATPase acting as a sensor of amino acid levels in the lysosome membrane adjacent to Rag GTPases and the Ragulator complex. In presence of high amino acid levels, Rag GTPases are activated and induce mTORC1 delocalization to the lysosomal membrane and thus its activation 128,129. A study of Mülleder *et al*. analyzed a yeast cell system, *Saccharomyces cerevisiae*, its amino acid metabolome and the effect of gene deletion. The main aim of this study was to determine the yeast biosynthetic regulome, using functional metabolomics. Yeast cells were collected by centrifugation and extracted using 80°C hot ethanol containing isotope-labeled amino acid standards. The lysate was cleared by centrifugation and amino acids were analyzed using HILIC and a tandem MS system and compounds were identified by matching retention time and fragmentation. The outcome was, among others, that TORC1 inhibition in exponentially growing cells matches the interruption of endomembrane transport 130.

Several studies indicate that a combination of increased anti-aging pathways and reduced nutrient and growth-related signaling pathways result in lifespan extension via the induction of autophagy 114. In line with this, supplementation of amino acids leads to lifespan extension in *C. elegans* 131. Autophagy, in addition to being a cellular response to nutrient deprivation, is also activated upon failure in degradation of misfolded proteins, a hallmark of neurodegenerative diseases. Aiming at understanding the molecular causes for the neurodegenerative disease Amyotrophic lateral sclerosis (ALS), Valbuena *et al*. carried out a metabolomics study of a well-characterized murine neuronal cell model of familial ALS expressing wild-type or mutant (G93A) superoxide dismutase (SOD) 132. Mutations in the gene of SOD are causative for familial forms of the neurodegenerative disease ALS 133,134. To investigate global effects of this mutant variant of SOD, untargeted metabolomics using NMR spectroscopy and GC-MS was used. Cells were cultured with either ^13^C glucose or ^13^C glutamine and metabolic flux was investigated. Cells were harvested in ice-cold methanol, dried, and intracellular metabolites were extracted and derivatized prior to GC-MS. Culture media were directly measured by NMR spectroscopy including an internal standard. Metabolomics data were analyzed using MatLab software packages. This study revealed increased lactate production in SOD G93A expressing cells upon serum deprivation. Increased levels of newly generated glycolysis and glutaminolysis products, but lower amino acid levels, were detected in SOD G93A expressing cells. This amino acid deprivation suggests impaired autophagy in SOD G93A expressing cells since cells deficient in autophagy are unable to maintain amino acid levels 132.

Shen *et al*. carried out a study on a murine cell model (MEFs) in order to characterize the importance of Atg7. The main aim of this study was to elucidate the metabolite profile of Atg7-dependent autophagy by comparing metabolism in Atg7^-/-^ and wild-type MEFs under acute starvation 52. Wild-type and Atg7^-/-^ cells were cultured and, after starvation, scraped in cold methanol. In order to quench intracellular metabolism, cells were frozen in liquid nitrogen and further metabolite extraction was performed 30 minutes at -20°C. The supernatant was used for UPLC-Q-MS analysis with a mass spectrometer operating in positive electrospray ionization. This study included a quality control sample by mixing equal volumes of each sample in order to obtain a mean profile for all analytes encountered during analysis. Metabolite identification was performed using databases (i.e. HMDB, METLIN) and verified by chemical standards with exact m/z values. SIMCA was used to get PCA scores and metabolic pathway analysis (MetPA by Metabolanalyst 3.0) helped to identify metabolites significantly altered upon starvation. PCA analysis revealed significant differences between wild-type and Atg7^-/-^ MEFs. 18 altered metabolites under starvation in wild-type, and 19 altered metabolites under starvation in Atg7^-/-^ were identified, and, within these metabolites, seven showed aberrant patterns in wild-type and Atg7^-/-^. These altered metabolites indicate a disturbance of amino acid, energy, lipid and nucleotide metabolism under starvation in wild-type MEFs, whereas amino acid, carbohydrate and energy metabolism were affected in Atg7^-/-^ MEFs. In summary, wild-type MEFs showed an increased lipid metabolism, delaying cell death. After four hours of starvation, apoptosis increased, whereas autophagy decreased, which affected amino acid, carbohydrate and energy metabolism. Contrary, in Atg7^-/-^ MEFs, due to their autophagy-deficiency, only apoptosis was occurring. These data underlined the importance of Atg7 in autophagy in response to acute starvation.

### Lipids

Elevated levels of free fatty acids or triglycerides are linked to induction of autophagy 115. For instance, palmitate induced autophagy requires mitogen-activated protein kinase 8 (MAPK8). Hence, despite being nutrients, lipids can induce autophagy, which may constitute an important mechanism to avoid potential lipotoxicity. Moreover, the need of lipids in autophagosome formation may implicate an induction of autophagy in presence of high lipid levels 51,115. Metabolomics studies of the complex class of lipids is typically carried out using MS-based technology, which provides information on individual lipid species, although NMR spectroscopy can provide quantitative information on lipid classes 81,135,136,137. One major activator of autophagy in the class of lipids is phosphatidylinositol-3-phosphate (PI3P) 138,139. High levels of unsaturated fatty acids as well as certain saturated fatty acids (mainly C15-C18) are capable of inducing autophagy. In general, it is known that the saturated fatty acids (C15-C18) induce autophagy via production of PI3P, whereas unsaturated fatty acids induce non-canonical autophagy. Production of PI3P is mediated by activation of phosphatidyl-inositol 3-kinase catalytic subunit type 3 (PIK3C3), which can in further consequence convert phosphatidylinositol (PI) to PI3P. Based on the knowledge of autophagy-regulation via lipids, a study by Enot *et al*. aimed at determining metabolic effects of autophagy-inducing doses of oleate and palmitate in a mouse model with respect to pro- or anti-autophagic metabolites. *In vivo* mouse models were intraperitoneally administered a single dose of palmitate or oleate, and the metabolic disturbances were analyzed. Hereby, metabolic profiles in tissues were detected in liver, heart and skeletal muscle. Tissues were homogenized using beads, and dried extracts were re-suspended in methanol and used for GC- and LC-MS. Data analysis was performed using a quantitative analysis software, and statistical analysis was performed using free available statistics software R 138,139. Depletion of amino acids, spermidine and spermine in the liver was observed after palmitate administration, whereas oleate induced an increase of NAD^+^. Moreover, palmitate raised acyl-carnitine levels in the heart. Overall, this study revealed an increase of anti-aging metabolites by palmitate, but not by oleate.

Induction of autophagy is mediated via reduction of p53 expression. Loss of tumor suppressor p53 in presence of docosahexaenoic acid (DHA) leads to activation of AMPK, and, in turn, inhibition of mTORC activity 140,141. DHA is a metabolite which can be investigated using, for instance, GC-MS, and provide information on the autophagic flux, which is increased in presence of DHA 142.

### Metabolic regulation of protein acetylation

Protein acetylation is mediated by lysine acetyltransferases by transferring an acetyl-group from acetyl-CoA to a lysine residue in the polypeptide chain. This regulatory acetylation can, on the one hand, regulate functions of cellular proteins by removing positive charges, and, on the other hand, modify gene transcription by reducing the affinity of histones to the phosphate backbone of DNA 117. Increased acetylation in cells can negatively regulate autophagy due to the increased rate of transcription and modified protein functions. Atg proteins are, in their de-acetylated state, capable of inducing formation of autophagosome and autophagy. In the acetylated state, Atg proteins do not induce autophagy 143. Upregulation of protein acetylation can be measured directly by proteomics 144 and indirectly by determination of acetyl-CoA levels. High cellular acetyl-CoA levels indicate better substrate availability for lysine acetyltransferases and therefore a higher level of protein acetylation, which is associated with reduced autophagy 46,114,117,145,146. Acetyl-CoA is a metabolite mainly detected by LC-MS due to its low abundance, while the associated metabolite acetate is well accessible by NMR spectroscopy 147. Apart from acetyl-CoA other metabolites, directly or indirectly related to protein acetylation, including nicotinamide adenine dinucleotide (NAD^+^), spermidine or hydroxybutyrate, are accessible, using both, NMR spectroscopy and MS 148,149,150.

NAD^+^ and their interaction partners, the sirtuin proteins, have initially been linked to reduced autophagy in protein misfolding diseases. Sirtuins, functionally known as protein deacetylases, are biomolecules capable of removing acetyl residues from proteins in a NAD^+^-dependent manner. Elevated concentrations of the cofactor NAD^+^ activate sirtuin deacetylase proteins, thus reducing protein acetylation. In case of Atg proteins, deacetylation enhances autophagosome formation and therefore autophagy 151,152,153. In protein folding diseases removal of misfolded or truncated proteins from the cellular environment through autophagy is impaired 154,155. Neurodegenerative diseases, in particular, have been linked to defective protein folding and in further consequence formation of intracellular proteinaceous inclusions. These abnormal protein aggregates may remain due to lack or inefficient autophagic rescue events 156,157,158,159,160. Indeed, reduced NAD^+^ levels have been associated with the exposure of cells to toxic misfolded prion protein 161. In line with this, exposure to NAD^+^ causes a decrease in mitochondrial content by activating autophagy via sirtuin activation 118,162,163.

These studies indicate that NAD^+^ concentrations are an important measure of autophagic flux, in biological systems. Increased NAD^+^ concentrations activate NAD^+^-dependent sirtuin deacetylase proteins and therefore reduce protein acetylation. NAD^+^, but also metabolites related to NAD^+^ metabolism (i.e. NADP^+^), are small molecules, which have been detected and quantified in different biofluids using targeted and untargeted NMR spectroscopy and MS-based methods 150,164,165,166.

A recently emerging compound associated with regulation of protein acetylation is spermidine. Spermidine is a small molecule polyamine and is an intermediate in the reaction of putrescine to spermine. It has been reported to decline during aging 167,168 and to regulate autophagy 46,167,169,170. Spermidine has been shown to regulate autophagy through inhibition of histone acetyltransferases 46,129,169, which results in hypoacetylated histone proteins. Acetylation of histones is a regulatory mechanism in gene transcription, which is not directly linked to autophagy. However, it can still, via up- or downregulation of autophagy-related genes, lead to regulatory modulation of autophagy. Spermidine can be detected by NMR spectroscopy and MS 46,170,171.

In a study by Eisenberg *et al*., the main focus was to reveal the role of spermidine in autophagy and aging in several *in vivo* systems, including yeast, flies, worms, human immune cells, and mice 172. Spermidine was applied to chronologically aging model systems and revealed a retard in cellular and organismal aging in all species. In line with this, a depletion of polyamines decreased the lifespan of yeast and induced necrosis. Furthermore, these studies revealed a link of lifespan extension to epigenetic hypoacetylation, which is ascribed to inhibition of histone acetyltransferase activity by spermidine. In order to quantify spermidine and other polyamines isolated from yeast cells, mouse liver tissues or flies after treatment of these model systems, the authors used a targeted LC-MS/MS approach. Extraction of polyamines from yeast was performed using an extraction procedure with trichloroacetic acid; polyamines of flies and from mouse liver tissue were extracted using freeze-thaw cycles. For LC-MS/MS measurements, a hydrophilic interaction liquid chromatography column was used and polyamines (spermidine, putrescine, bis(hexamethylene)-triamine) were identified based on their characteristic mass-over-charge ratio transitions of precursor to product fragment ions and retention times (multiple reaction monitoring). For quantification, calibration standards were prepared by spiking extraction buffer with specific concentrations of spermidine, putrescine and an internal standard 172.

Autophagy is a catabolic process, which helps cells and cellular organisms to cope with stress situations. Oxidative stress has also been linked to autophagy 173, and oxidative stress response is strictly regulated. Transporter of polyamines 1 (Tpo1) controls intracellular spermine and spermidine concentrations, as well as the induction of antioxidant proteins. In a study by Krüger *et al*. the main focus was on adaptions to oxidative stress with respect to polyamine transport in eukaryotic yeast cells. Their aim was to determine whether export of spermine and spermidine influences adaption to unfavorable environmental conditions, using *S. cerevisiae* as a model system. By applying oxidative stress via H_2_O_2_ exposure, the export of polyamines via Tpo1 and expression of antioxidant proteins was induced. In order to determine the amount of polyamines in cells, putrescine, spermine and spermidine levels were quantified using a targeted LC-MS/MS approach. For extraction, yeast cells were harvested, washed and homogenized using glass beads on a homogenizer. After centrifugation, the supernatant was used for derivatization and subsequently chromatographically separated. Identification of polyamines was obtained via their characteristic mass-over-charge ratio transitions of precursor to fragment ions of polyamines (multiple reaction monitoring) 170.

Most of the discussed studies included only a small number of metabolites, which indicates that there is still an enormous potential for metabolomics research in autophagy. Until now, we can refer to these aforementioned studies, but due to the current powerful state-of-the-art techniques in metabolomics and the emerging research in this field, the number of autophagy studies using metabolomics can be expected to increase in future. For these studies, any autophagy-related setting ranging von knockout of Atg proteins 49,52, to treatment with inhibitors 54 or starvation 49,52 can be used in order to study metabolome of biological samples. Samples can be analyzed as long as they are soluble (or volatile in case of GC-MS), and high enough concentrated for the respective technique. This enables almost infinite capabilities of studying metabolism in autophagy.

## CONCLUDING REMARKS

Conceived in general terms, autophagy is a process which is regulated by unfavorable environmental conditions, including stresses and starvation. These conditions lead to metabolic disturbances and aberrations which can be determined in biological samples using NMR- and MS-based metabolomics. Quantitative information for key regulatory metabolites, including glucose, amino acids, fatty acids, acetyl-CoA, NAD^+^, spermidine and many other, provide detailed molecular insight into autophagic processes 48,111,118,129,131,141,145. Therefore, metabolomics is a powerful technique, which can, despite the high complexity of autophagic processes in biological systems, help to further understand and characterize distinct pathways. Apart from the autophagy-related proteins, metabolites are compounds that regulate autophagy or reflect autophagic processes in cell systems 46,129,173. Due to high throughput and robust detection in metabolomics, it enables characterization of a high number of metabolites in large sample series with reliable results. Information gathered in these studies will further improve current knowledge about autophagy, its regulation and its outcome 11,12,53,174.

Recent technological developments have enabled metabolomics research in a plethora of biological matrices, including, but not limited to cell and tissue lysates, and biofluids, and have established NMR- and MS-based metabolomics as the key techniques in clinical research. Given their broad applicability and the systemic insights into metabolism that is obtained it is not surprising that NMR- and MS-based metabolomics became increasingly important in basic biological research. It can be expected that metabolomics will establish itself as a standard technique in basic biomedical research in the near future.

It can also be anticipated that MS and NMR-based metabolomics will be further integrated with other techniques. For example, NMR is excellently suited as a proxy for *in vivo* magnetic resonance metabolite imaging. Metabolite information, such as resonance frequencies and concentrations, derived from NMR-based metabolomics guide the setup of *in vivo* magnetic resonance metabolite imaging studies and allow tracking metabolite localization and concentration in real time in living organisms. Hu *et al*. performed a study on Myc, a protein impairing autophagosome formation, using *in vivo* metabolite imaging 175. Since the temporal relationship between oncogene signaling, *in vivo* tumor formation and glycolytic pathway activity is not understood so far, their main aim was to reveal the onset of metabolic changes in *de novo* tumor formation using ^13^C pyruvate. Using these experiments, the authors discovered altered glycolysis, namely a predominant conversion of pyruvate to alanine in pre-cancerous tissues before observing histologic or morphologic changes 2,82,175. This study indicates, that in the future *in vivo* magnetic resonance imaging may enlighten key metabolic pathways involved in autophagy. Similar MS-based imaging approaches enable determination of metabolite distribution in tissue sections of whole-body or single heterogeneous organ samples 176. Innovative cell-sampling technologies and highly sensitive mass spectrometry allow even metabolic profiling in single cells, and can be combined with microfluidics 31,177. In addition, integrative approaches for multi-omics data analysis will provide the outmost information content in biological studies 178,179,180.

A large number of autophagy studies analyzes the effect of starvation/knockout of Atg proteins by reducing the supply of i.e. glucose and investigating effects on protein level, mRNA level, via histology or via microscopy 173,181,182,183. These studies provide an indirect information about metabolic changes in cells, tissues or organisms. However, they do not clearly depict the metabolism of the samples. By additionally applying metabolomics in order to investigate effects of these autophagy-settings, the information content will be enormously increased. In the future, metabolomics will be applied in many different fields, which will make the automation of all processes, including sample preparation, measurement and data analysis, inevitable.

There are several open questions in autophagy research, which makes it an important and emerging research field. For instance, it is not clear yet whether fluctuations in the abundance of specific metabolites might stimulate a specific and graduated autophagic response 115. In addition, it remains elusive what makes the cells ‘know’ if autophagy is efficient or not 184. Finally, the switch of autophagy facilitating cell health to autophagy promoting programmed cell death is not understood so far 185. These are only some of the open questions that might be successfully addressed by metabolomics techniques.

Summarizing, NMR spectroscopy and MS are well suited for in-depth metabolomic analysis and well-applicable to study the molecular mechanisms involved in autophagy. By combining both techniques a large metabolic space is covered. Given the recent success of metabolomics it can be expected that metabolomics approaches will contribute significantly to deciphering the complex regulatory mechanisms involved in autophagy in the near future and promote understanding of autophagy and autophagy-related diseases in living cells and organisms.
